# Simulation-based Mastery Learning Improves Emergency Medicine Residents’ Ability to Perform Temporary Transvenous Cardiac Pacing

**DOI:** 10.5811/westjem.2022.10.57773

**Published:** 2022-12-28

**Authors:** Matthew R. Klein, Zachary P. Schmitz, Mark D. Adler, David H. Salzman

**Affiliations:** * Northwestern University Feinberg School of Medicine, Department of Emergency Medicine, Chicago, Illinois; † NYU Grossman School of Medicine, New York City, New York; ‡ Northwestern University Feinberg School of Medicine, Department of Pediatrics, Department of Medical Education, Chicago, Illinois; § Northwestern University Feinberg School of Medicine, Department of Medical Education, Chicago, Illinois

## Abstract

**Introduction:**

Temporary transvenous cardiac pacing (TVP) is a critical intervention that emergency physicians perform infrequently in clinical practice. Prior simulation studies revealed that emergency medicine (EM) residents and board-certified emergency physicians perform TVP poorly during checklist-based assessments. Our objective in this report was to describe the design and implementation of a simulation-based mastery learning (SBML) curriculum and evaluate its impact on EM residents’ ability to perform TVP.

**Methods:**

An expert panel of emergency physicians and cardiologists set a minimum passing standard (MPS) for a previously developed 30-item TVP checklist using the Mastery Angoff approach. Emergency medicine residents were assessed using this checklist and a high-fidelity TVP task trainer. Residents who did not meet the MPS during baseline testing viewed a procedure video and completed a 30-minute individual deliberate practice session before retesting. Residents who did not meet the MPS during initial post-testing completed additional deliberate practice and assessment until meeting or exceeding the MPS.

**Results:**

The expert panel set an MPS of correctly performing 28 (93.3%) checklist items. Fifty-seven EM residents participated. Mean checklist scores improved from 13.4 (95% CI 11.8–15.0) during baseline testing to 27.5 (95% CI 26.9–28.1) during initial post-testing (P < 0.01). No residents met the MPS at baseline testing. The 21 (36.8%) residents who did not meet the MPS during initial post-testing all met or exceeded the MPS after completing one additional 30-minute deliberate practice session.

**Conclusion:**

Emergency medicine residents demonstrated significantly improved TVP performance with reduced variability in checklist scores after completing a simulation-based mastery learning curriculum.

## INTRODUCTION

Temporary transvenous cardiac pacing (TVP), a potentially life-saving intervention for critically-ill patients with unstable bradycardia,^1^ is a vital component of the emergency physician’s skillset but is performed infrequently in clinical practice.^2^ Although the Accreditation Council for Graduate Medical Education (ACGME) includes cardiac pacing in its list of key index procedures that are essential for the independent practice of emergency medicine (EM),^3^ residency programs cannot guarantee every trainee an opportunity to perform TVP during patient care. Unsurprisingly, limited exposure to TVP during training raises concerns about procedural competence,^4^,^5^ and EM graduates feel underprepared to perform TVP.^6^ Checklist-based assessments conducted in a simulation setting reveal poor baseline TVP performance,^7^ with both EM residents and board-certified emergency physicians correctly performing, on average, fewer than half of the necessary steps of this critical procedure.^8^,^9^

This reported lack of confidence and objective poor performance suggest the need for more effective models of TVP training during EM residency. Simulation provides an opportunity for learners to demonstrate procedural competence in a controlled setting.^10^ Mastery learning is a form of competency-based education in which all learners meet fixed achievement standards after individualized and variable amounts of educational time.^11^ Simulation-based mastery learning (SBML) is a rigorous educational paradigm that employs an integrated bundle of seven features^12^ (Table 1) and produces superior learning outcomes compared to non-mastery instruction.^13^,^14^ SBML is a proven method for assessing and improving trainee performance for a variety of invasive procedures in the EM scope of practice, including lumbar puncture,^15^ central venous catheter insertion,^16^ and emergency department (ED) thoracotomy.^17^ SBML seems well-suited to improving TVP training and performance; however, to our knowledge no mastery learning curriculum exists for this procedure.

Our goal in this report was to describe the design and implementation of an SBML curriculum for TVP. Specific objectives included first establishing a minimum passing standard (MPS) for this procedure by consensus of an expert panel using the Mastery Angoff approach.^18^ Additional objectives included determining whether this curriculum led to achievement of the MPS by all learners and comparing baseline and post-training checklist scores for EM residents performing TVP in a simulated environment. We also present program evaluation data.

**Population Health Research Capsule**

What do we already know about this issue?

*Transvenous pacing (TVP) is a potentially life-saving intervention, but opportunities for emergency medicine (EM) residents to develop competence with TVP are limited.*

What was the research question?

*Can a simulation-based mastery learning curriculum improve EM residents’ ability to perform TVP?*

What was the major finding of the study?

*Mean checklist score improved from 13.4/30 to 27.5 during initial post-testing. All learners reached mastery.*

How does this improve population health?

*After completing this curriculum, all learners demonstrated the ability to perform this rare but potentially life-saving procedure.*

## METHODS

### Study Design and Setting

**Figure 1:**
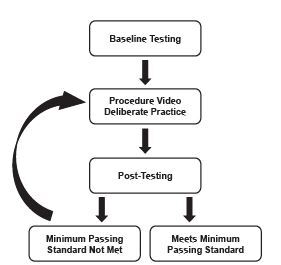
Flow diagram showing the study design using the mastery learning model.^19^

Like previously described SBML interventions,^17^ this prospective, cohort study used a pretest-post-test design following the mastery learning model (Figure 1).^19^ We conducted this study at a single, urban, academic medical center with a four-year EM residency during the 2019–2020 academic year. The institutional review board reviewed this study and determined it to be exempt.

All learners completed baseline diagnostic testing during a five-week period in October–November 2019. Those who did not demonstrate mastery during baseline testing by meeting or exceeding a predetermined MPS were required to complete an educational intervention, which took place between December 2019–February 2020. The intervention consisted of viewing a procedure video followed by deliberate practice with individual feedback. After the intervention, learners were again assessed using the checklist from February–April 2020; those who did not meet or exceed the MPS at initial post-testing completed additional deliberate practice and additional post-testing in May–June 2020 until all learners met or exceeded the MPS. Learners evaluated the curriculum after initial post-testing.

### Selection of Participants

All postgraduate year (PGY) 1–4 EM residents at our institution were eligible for inclusion in this study. One resident was excluded due to participation in the study design and assessment.

### Intervention

We created a series of learning objectives with increasing complexity for this curriculum. These included describing the indications and contraindications for TVP, listing the necessary equipment, demonstrating each step of the procedure, and then performing TVP to a mastery standard on a high-fidelity task trainer as assessed by a checklist. The first component of the educational intervention was an 11-minute video that we scripted and which was then filmed by professional videographers. The video begins with a review of indications, contraindications, relevant anatomy, and necessary equipment for TVP. Next, the video provides a narrated demonstration of each step of the procedure as detailed in the checklist. To encourage attention to the overall procedural skill and avoid rote memorization of the checklist itself, individual checklist items are not identified or displayed. The video was stored on a password-protected website and was shared only after all learners completed baseline testing. Learners were required to view the video prior to their deliberate practice session, and there was no limit on the number of times the video could be reviewed.

The second component of the educational intervention consisted of an individual, 30-minute session for each learner, designed to follow principles of deliberate practice (ie, an interactive discussion based on directly observed performance, using a “pause and discuss” model as events unfold).^20^ These sessions were all led by a single instructor working with one learner at a time and followed a structured format to ensure consistency in session content. Each session began with a review of the checklist items most commonly not performed or performed incorrectly by all learners. Checklist items that were not performed or performed incorrectly by the individual learner were then reviewed, and correct performance was demonstrated using the simulator. Learners were able to practice these items as many times as necessary until correct performance was demonstrated. Finally, each learner practiced the entire procedure from beginning to end while receiving directly observed coaching and feedback.

### Measurements

Learners were assessed using a 30-item, dichotomous (“correct” vs “incorrect/not done”) checklist created for this curriculum with input from emergency physicians, interventional cardiologists, and electrophysiologists. The checklist prompted learners to perform TVP in a patient with unstable bradycardia in whom an introducer sheath had already been placed. (During checklist development, the expert panel decided that placing the introducer sheath was a separate procedure.) A detailed description of the checklist’s design, content, and characteristics was previously reported, along with data demonstrating strong interrater reliability for checklist scores and the ability for the checklist to distinguish learners at different levels of training.^8^

A panel of 12 board-certified physicians (two cardiologists and 10 emergency physicians with experience performing and teaching TVP) established an MPS for the checklist using the Mastery Angoff approach.^18^ Panelists were recruited from our professional network and represent a diversity of geographic practice areas. None of the panelists participated in other aspects of the curriculum, including checklist design. One author facilitated the standard setting by leading a video conference call with the panelists that began by describing the purpose of the curriculum, the intended learners, and the concept of mastery learning. The checklist was then reviewed in detail.

Consistent with the Mastery Angoff approach, panelists were asked to estimate the percentage of well-prepared learners (defined as an individual who could safely and successfully perform the procedure in clinical practice with minimal or no supervision) who would correctly perform each checklist item after completing the curriculum. Panelists finalized their estimates after a group discussion and submitted their responses electronically. Each panelist’s individual MPS was determined by averaging their individual checklist item percentages. We then averaged these individual standards to determine the overall MPS for the checklist.

All learners were assessed by the same rater who stood adjacent to the task trainer and scored learners’ performance using a web-based version of the checklist (Qualtrics LLC, Provo, UT). The rater was blinded to the MPS until completion of the study. For compound items (for example, item 10: “Attaches the cap to the introducer sheath and turns the cap to lock into place on the sheath”), the item was scored as correct only if the learner performed all actions described in the item. To ensure all learners had the opportunity to demonstrate item 30 (“Retracts pacing wire only when balloon is deflated throughout procedure”), the TVP software was programmed so that successful capture could not be obtained on the first attempt. The same method was used for baseline assessment and post-testing. Learners did not have access to the checklist at any point during the curriculum.

We collected course evaluation data using an electronic survey (Qualtrics) following initial post-testing. Survey questions focused on whether learners felt the curriculum was a beneficial addition to their residency training, their enjoyment of the session, and whether learners felt confident performing TVP in the ED. Responses were scored on a Likert scale 1–5 (1 = strongly disagree; 5 = strongly agree).

### Outcomes

The primary outcome measure was the mean number of correctly performed checklist items at each phase of the curriculum (baseline testing, initial post-testing, additional post-testing). Secondary outcome measures included program evaluation data obtained from a post-curriculum survey.

### Analysis

Individual learner performance was determined by calculating the total number of correctly performed checklist items. Mean scores for all learners are reported with 95% confidence intervals (CI). We used the Wilcoxon signed-rank test to evaluate the difference in scores between baseline and initial post-testing. For program evaluation data, we calculated mean Likert scores with standard deviations. All analysis was performed using Stata 16.1 (StataCorp, College Station, TX).

## RESULTS

### Characteristics of Study Subjects

There were 59 residents eligible for inclusion in this study; two PGY-4 residents were unable to participate in the study due to scheduling conflicts. We collected data for 57 participants, including 15 PGY-1s, 15 PGY-2s, 15 PGY-3s and 12 PGY-4s. There were 38 (66.7%) male and 19 (33.3%) female residents enrolled.

### Main Results

**Figure 2:**
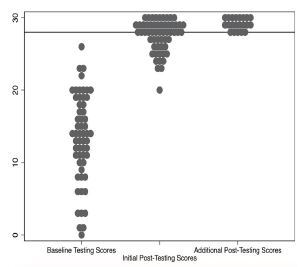
Distribution of checklist scores during each phase of testing. Vertical axis depicts checklist score (maximum score 30). Horizontal line represents the minimum passing standard (MPS) of 28/30. Additional post-testing scores represent the 21 learners who did not meet the MPS during initial post-testing.

The expert panel set an MPS of correctly performing 28 (93.3%) checklist items. Performance data is displayed in Figure 2. No learners met the MPS during baseline testing. The mean score at baseline testing was 13.4 (95% CI 11.8–15.0). The mean score at initial post-testing was 27.5 (95% CI 26.9–28.1). The difference between the mean score at baseline testing and initial post-testing was statistically significant (*P* < 0.01).

Of the 57 residents enrolled, 36 (63.2%) achieved the MPS during initial post-testing. The mean score for the 21 residents (eight PGY-1s, six PGY-2s, five PGY-3s, and two PGY-4s) who did not meet or exceed the MPS during initial post-testing was 25.2 (95% CI 24.4–26.0). After completing one additional 30-minute, deliberate practice session, each of these 21 residents met or exceeded the MPS during additional post-testing. Checklist items that were most commonly not performed or performed incorrectly during baseline testing included the following: confirming the connection between the pacing wire and 2-millimeter adapters; confirming mechanical capture; securing the pacing generator after the procedure; and locking the introducer sheath cap O-ring. Complete data for checklist performance is presented in Table 2.

Course evaluations were completed by 51 (89%) of the participants. Learners indicated that the curriculum was a beneficial (mean 5.0, SD 0.2) and enjoyable (mean 4.9, SD 0.3) addition to their training, and they felt confident (mean 4.6, SD 0.6) performing TVP in the ED after completing the course.

## DISCUSSION

This report describes the design and implementation of a SBML curriculum for training EM residents to perform TVP. After viewing a procedure video and completing individualized amounts of deliberate practice, all learners met or exceeded the MPS set for this procedure. These findings add to existing literature that shows SBML to be a highly effective form of competency-based medical education, particularly for infrequently performed invasive procedures.^15^–^17^ The rigorous MPS set for this curriculum – 93.3% – is similar to the high standards set for other mastery learning interventions.^18^,^21^

While mastery, defined as meeting the MPS, is ultimately a dichotomous variable, analyzing performance by checklist score allowed for a more granular understanding of a learner’s skill before and after undergoing the educational intervention. The poor baseline performance in this cohort – a mean score of 13.4 on a 30-item checklist – is not surprising for such an uncommon intervention and is consistent with the poor baseline performance observed in prior mastery learning studies of other procedures.^15^–^17^ Previous TVP training in our program did not follow a competency-based model, and a majority of our residents had not performed TVP on a patient prior to the beginning of this curriculum; we suspect this limited experience is similar to that of trainees at other EM programs.

All learners in this report demonstrated the ability to meet or exceed the MPS after completing this curriculum, which included rigorous assessment, an educational intervention (watching the procedure video), and individualized amounts of deliberate practice with directly observed coaching and feedback, which are essential features of mastery learning. Although the mean checklist score improved significantly from baseline testing to initial post-testing, for more than one third of the learners, a single deliberate practice session did not result in meeting or exceeding the MPS. This is not unexpected. In mastery learning there is no “failure,” only the opportunity for additional practice and testing until all learners achieve the desired standard.

The mean checklist score for the learners who required additional practice was still notably improved from initial post-testing, with a narrower range of performance. Following one additional 30-minute, deliberate practice session, these learners all met or exceeded the MPS during additional post-testing. Despite the additional time commitment required to complete this curriculum, learners found it to be an enjoyable and beneficial addition to their training and felt confident in their ability to perform TVP after completing the course.

Procedural competence is critical to the practice of EM^22^,^23^; however residency programs cannot guarantee trainees exposure to all procedures in the clinical environment prior to graduation.^24^,^25^ This is particularly true for TVP, which, due to its rarity, is one of only three procedures for EM allowed by the ACGME to be exclusively performed through simulation prior to residency graduation.^3^ As growing numbers of learners in all specialties compete for access to a finite number of invasive procedures in teaching hospitals, clinical experience alone may no longer be sufficient to ensure procedural competence.^26^ Notably, the 21 learners in this study who did not meet the MPS during initial post-testing included PGY-1 through PGY-4 residents, which suggests that skill level and need for additional practice cannot be predicted solely by level of training. Rigorous, competency-based training and assessment methods like mastery learning can – and should – be used to prepare EM residents to successfully and independently perform uncommon but potentially lifesaving interventions.

## LIMITATIONS

There are several limitations to this report. A relatively small number of learners completed the curriculum due to the single-site design; a larger sample size could improve the generalizability of these findings. Prior to the creation of this SBML intervention, our residents completed TVP training during an annual “rare procedures” simulation session that did not follow a competency-based model and which residents attended as their clinical schedule allowed; we acknowledge that learners with alternate methods of prior training may demonstrate different levels of performance in this curriculum.

We were unable to track how many times the procedure video was viewed; however, all learners verbally attested to watching the video prior to their deliberate practice session. Finally, this curriculum required a significant investment of time and resources: learners participated in a minimum of three sessions (baseline testing, deliberate practice, and initial post-testing), each of which required direct observation and active involvement of a faculty member. Specialized equipment and support from simulation staff was also required. The resource-intensive nature of mastery learning may limit its feasibility in other settings.

Given the small sample size, we did not compare SBML to other methods of TVP training. However, mastery learning has been consistently shown to outperform non-mastery instruction.^13^–^15^,^27^ In addition, we did not control for learners who had previous experience performing TVP during clinical practice. However, no learners in our cohort demonstrated mastery during baseline testing, which indicates that any residents with prior clinical TVP experience were not adequately prepared to meet or exceed the MPS set for this procedure. This is consistent with a previous analysis of other invasive procedures performed by internal medicine and EM residents (including central venous catheter insertion, lumbar puncture, and thoracentesis), which found that clinical procedure experience during residency was not sufficient to ensure competence.^28^

Future research should investigate the optimal timing for repeat assessment and practice to ensure TVP skill retention. Assessment of other specialists who perform TVP, such as cardiologists and intensivists, should be explored. While challenging to assess, the effect of this curriculum on patient-level outcomes (ie, Kirkpatrick Level 3) should also be investigated. Finally, the deliberate practice and assessment components of this curriculum were conducted using a high-fidelity simulator; adapting the curriculum to incorporate low-fidelity models could also be explored.^29^

## CONCLUSION

This study demonstrated a significant improvement in emergency medicine residents’ ability to perform temporary transvenous cardiac pacing on a high-fidelity task trainer after completing a mastery learning curriculum and contributes to the growing body of literature that proves simulation-based mastery learning to be a highly effective method of competency-based medical education.

## Figures and Tables

**Table 1:** Seven features of a mastery learning bundle.^12^

Baseline diagnostic testing Clear learning objectives sequenced as units ordered by increasing difficulty Engagement in educational activities (eg, deliberate skills practice, coaching, data interpretation, reading) that are focused on reaching the objectives The establishment of a minimum passing standard (e.g., test score, checklist score) for each educational unit Formative testing with actionable feedback to gauge unit completion, or the need for more practice at the preset minimum passing standard Advancement to the next educational unit given measured achievement at or above the minimum passing standard Continued practice or study on an educational unit until the minimum passing standard is reached

**Table 2:** Learner performance for individual checklist items.

Checklist item		Baseline testing*		Initial post-testing		Additional post-testing	
		n	(%)	n	(%)	n	(%)
1	Gathers necessary equipment	10	17.5%	43	75.4%	21	100.0%
2	Cleans hands	19	33.3%	48	84.2%	20	95.2%
3	Dons personal protective equipment	42	73.7%	55	96.5%	21	100.0%
4	Identifies the appropriate syringe for balloon inflation and fills syringe with 0.75 mL of air and attaches to the port of the pacing wire	37	64.9%	56	98.2%	20	95.2%
5	Assesses the patency of the balloon	38	66.7%	57	100.0%	21	100.0%
6	Attaches the 2-mm adapters to the proximal end of the pacing wire and confirms integrity of connection by gently tugging on connectors	1	1.8%	50	87.7%	21	100.0%
7	Hands the distal end of the pacing wire (with 2-mm adapters attached) and syringe to non-sterile assistant	37	64.9%	57	100.0%	21	100.0%
8	Instructs non-sterile assistant to attach the 2-mm adapters to the pacing generator adapter cable and confirms integrity of connection by gently tugging on the attached wires	4	7.0%	43	75.4%	18	85.7%
9	Asks non-sterile assistant to attach the cable to the pacing generator	46	80.7%	56	98.2%	21	100.0%
10	Attaches the cap to the introducer sheath and turns the cap to lock into place on the sheath	14	24.6%	56	98.2%	20	95.2%
11	Inserts the distal tip of the pacing wire through the sterile sheath in the correct orientation	46	80.7%	55	96.5%	21	100.0%
12	Inserts the pacing wire through the introducer sheath to at least the 20-cc mark	51	89.5%	55	96.5%	21	100.0%
13	Inflates the balloon and ensures the stopcock is closed after balloon inflation	32	56.1%	53	93.0%	20	95.2%
14	Asks non-sterile assistant to turn on the pacing generator	46	80.7%	57	100.0%	21	100.0%
15	Asks non-sterile assistant to select a mode that allows for ventricular pacing	43	75.4%	57	100.0%	21	100.0%
16	Asks non-sterile assistant to select a rate of 60–80 beats per minute on the pacing generator	44	77.2%	57	100.0%	21	100.0%
17	Asks non-sterile assistant to select a ventricular output of 10–25 mA (maximum) on the pacing generator	31	54.4%	57	100.0%	21	100.0%
18	Advances pacing wire with balloon inflated until electrical capture is achieved	27	47.4%	54	94.7%	21	100.0%
19	Asks non-sterile assistant to confirm mechanical capture by palpation of pulses	4	7.0%	42	73.7%	18	85.7%
20	Deflates balloon	14	24.6%	46	80.7%	20	95.2%
21	Turns the stopcock for the balloon to the OFF position	9	15.8%	40	70.2%	17	81.0%
22	Asks non-sterile assistant to decrease ventricular output amps until capture is lost	20	35.1%	52	91.2%	21	100.0%
23	Asks non-sterile assistant to increase ventricular output amps to a level higher than that which resulted in electrical capture	13	22.8%	50	87.7%	21	100.0%
24	Locks introducer sheath cap O-ring	9	15.8%	51	89.5%	20	95.2%
25	Extends sterile sheath to cover the length of the pacing wire and connects sterile sheath to introducer sheath cap	20	35.1%	55	96.5%	21	100.0%
26	Locks O-ring on the sterile sheath covering	21	36.8%	51	89.5%	21	100.0%
27	Secures pacing generator	6	10.5%	56	98.2%	20	95.2%
28	Orders chest radiograph to confirm location of pacing wire	7	12.3%	48	84.2%	20	95.2%
29	Maintains full sterility throughout procedure	48	84.2%	55	96.5%	21	100.0%
30	Retracts pacing wire only when balloon is deflated throughout procedure	24	42.1%	57	100.0%	21	100.0%

* The number (n) and percentage (%) of learners who performed each checklist item correctly during each phase of testing.
